# Epigenomic profiling of isolated blood cell types reveals highly specific B cell smoking signatures and links to disease risk

**DOI:** 10.1186/s13148-023-01507-8

**Published:** 2023-05-25

**Authors:** Xuting Wang, Michelle R. Campbell, Hye-Youn Cho, Gary S. Pittman, Suzanne N. Martos, Douglas A. Bell

**Affiliations:** Environmental Epigenomics and Disease Group, Immunity, Inflammation and Disease Laboratory, Intramural Research Program, National Institute of Environmental Health Sciences, National Institutes of Health, Research Triangle Park, NC 27709 USA

**Keywords:** DNA methylation, Tobacco smoking, Blood cell types, Epigenome-wide association study, Genetic variation, Memory B cell

## Abstract

**Background:**

Tobacco smoking alters the DNA methylation profiles of immune cells which may underpin some of the pathogenesis of smoking-associated diseases. To link smoking-driven epigenetic effects in specific immune cell types with disease risk, we isolated six leukocyte subtypes, CD14+ monocytes, CD15+ granulocytes, CD19+ B cells, CD4+ T cells, CD8+ T cells, and CD56+ natural killer cells, from whole blood of 67 healthy adult smokers and 74 nonsmokers for epigenome-wide association study (EWAS) using Illumina 450k and EPIC methylation arrays.

**Results:**

Numbers of smoking-associated differentially methylated sites (smCpGs) at genome-wide significance (*p* < 1.2 × 10^−7^) varied widely across cell types, from 5 smCpGs in CD8+ T cells to 111 smCpGs in CD19+ B cells. We found unique smoking effects in each cell type, some of which were not apparent in whole blood. Methylation-based deconvolution to estimate B cell subtypes revealed that smokers had 7.2% (*p* = 0.033) less naïve B cells. Adjusting for naïve and memory B cell proportions in EWAS and RNA-seq allowed the identification of genes enriched for B cell activation-related cytokine signaling pathways, Th1/Th2 responses, and hematopoietic cancers. Integrating with large-scale public datasets, 62 smCpGs were among CpGs associated with health-relevant EWASs. Furthermore, 74 smCpGs had reproducible methylation quantitative trait loci single nucleotide polymorphisms (SNPs) that were in complete linkage disequilibrium with genome-wide association study SNPs, associating with lung function, disease risks, and other traits.

**Conclusions:**

We observed blood cell-type-specific smCpGs, a naïve-to-memory shift among B cells, and by integrating genome-wide datasets, we identified their potential links to disease risks and health traits.

**Supplementary Information:**

The online version contains supplementary material available at 10.1186/s13148-023-01507-8.

## Background

Tobacco smoking leads to disease and disability and harms nearly every organ of the body. More than 16 million Americans are living with a disease caused by smoking (www.cdc.gov). Tobacco smoke has pro-inflammatory and immunosuppressive effects [1] and is a major environmental risk factor for adverse health outcomes including lung cancer, chronic obstructive pulmonary disease, cardiovascular disease, type 2 diabetes, tuberculosis, certain eye diseases, and problems of the immune system, including rheumatoid arthritis. At the cellular level, tobacco smoke exposure induces DNA damage [2] and influences mutation frequency [3–5]. Numerous recent epigenome-wide association studies (EWAS) using whole blood samples [6–15] have identified repeatable, smoking-associated DNA methylation sites (smCpGs) mapped to genes including aryl hydrocarbon receptor repressor (*AHRR*), growth factor independent 1 transcriptional repressor (*GFI1*), alkaline phosphatase, placenta like 2 (*ALPPL2*), Flotillin (*FLOT1*), and G protein-coupled receptor 15 (*GPR15*). However, the relatively small magnitude of methylation changes at any specific locus (usually < 10%), indicates that only small populations of cells in the blood are affected by the exposure.

Blood leukocytes display characteristic transcription, chromatin, and DNA methylation patterns associated with their immune functions [16]. Smoking is known to affect immune cell function [1] and composition [17], and epigenetic studies utilizing whole blood may be detecting changes in activated immune cell subsets [18, 19] or in specific leukocyte cell proportions. It is well recognized that these cell-type proportional changes may confound or affect the interpretation of results and useful algorithmic approaches for adjustment for cell-type changes have been developed [18, 20–26]. However, an adjustment may mask some useful information about the immune system, and detailed epigenetic studies assessing exposure effects on DNA methylation in specific cell types and relation to disease could help in understanding the meaning of whole blood-based EWAS results.

Smoking-related methylation changes in certain cell types could indicate different sensitivities to exposure and differing modes of action among cell lineages as well as potential functional effects that are important to cell-type-specific disease etiology or the early detection of disease. Our previous studies have examined smoking-associated methylation effects in purified CD14+ monocytes and observed associations with atherosclerosis markers [27] or upregulation of transcription via enhancer activation [28]. In a pilot study, we examined a limited set of CpGs across four cell types in a small group of smokers and observed differences in the response to smoking exposure among cell types [29]. The current study seeks to extend these observations to genome-wide smoking-driven epigenetic effects in six isolated blood cell types, utilizing the 12 cell-type adjustment model of Salas et al. [30] to differentiate naïve and memory cell types and to relate these to tobacco smoke-associated disease phenotypes.

We hypothesized that cell-type-specific alteration of DNA methylation profiles may underpin some of the pathogenesis of tobacco smoke-associated complex diseases by altering the capacity for immunological activation, differentiation, and other parameters that differ among leukocyte cell types. To explore this hypothesis, we isolated major cell types and assessed genome-wide DNA methylation with Illumina arrays in cell-type DNA obtained from adult smokers and nonsmokers. The use of the Salas et al. [30] model clearly reveals smoking-associated shifts in immune cell subtypes (naïve-to-memory transition) that likely drive smoking-altered methylation and transcription. By integrating smoking cell-type EWAS data with those from genetic studies (GWAS) of traits, phenotypes and transcription (expression quantitative loci, eQTLs, and methylation quantitative trait loci, mQTLs) in blood, we identify smCpGs associated with GWAS variants for a wide range of complex traits including lung function, demonstrating the utility of this approach for refining epigenetic association signals.

## Results

### Smoking impact on DNA methylation differs among isolated blood cell types

We isolated six major cell types from whole blood (WB) obtained from volunteer adult smokers (*n* = 67) and nonsmokers (*n* = 74) living in the Raleigh, Durham and Chapel Hill, North Carolina region using magnetic beads coated with antibodies against surface markers following the scheme shown in Additional file 1: Fig. S1 and determined genome-wide methylation levels using Illumina 450k and EPIC arrays. We applied a multivariable robust linear regression model [31] to adjust for potential confounding factors (Additional file 7: Table S1), including age, sex, ancestry, body mass index (BMI) and the presence of other cell types within a cell-type fraction as predicted by deconvolution algorithm [18]. Table 1 lists the counts of smoking-associated CpGs (smCpGs) and the proportion of CpGs that were specific to that cell type at different statistical significance levels, including genome-wide (Bonferroni, or BF, *p* < 1.2 × 10^−7^), and 5% false discovery rate (FDR 5%). Comparing EWAS carried out after winsorizing outliers (90% winsorization) in the DNA methylation dataset, smCpGs remaining at Bonferroni significance among cell types ranged from 91.8 to 100% (Additional file 7: Table S2).

**Table 1:** Distribution of significant and highly specific smoking-associated CpGs (smCpGs) among blood cell types and replication in published epigenome-wide association studies

Cell type (Sample n^a^)	CpGs at BF (Percent specific to this cell type)	Highly specific among BF (Percent highly specific)^b^	FDR 5% (Percent specific to this cell type)	BF replicated^c^	Replicated highly cell-type-specific^b^
B cell (CD19+) (NS = 69, SM = 64)^c^	111 (82.0%)	80 (72.1%)	3096 (93.4%)	62 (56%)	36 (45%)
Granulocyte (CD15+) (NS = 67, SM = 63)	96 (42.7%)	26 (27.1%)	986 (74.2%)	84 (88%)	16 (62%)
Monocyte (CD14+) (NS = 71, SM = 62)	83 (33.7%)	12 (14.5%)	501 (54.1%)	78 (94%)	10 (83%)
Natural killer cell (CD56+) (NS = 68, SM = 58)	19 (36.8%)	5 (26.3%)	482 (78.8%)	17 (90%)	4 (80%)
CD4T cell (CD4+) (NS = 67, SM = 60)	12 (66.7%)	5 (41.7%)	45 (62.2%)	12 (100%)	5 (100%)
CD8T cell (CD8+) (NS = 68, SM = 60)	5 (20.0%)	1 (20%)	10 (30.0%)	4 (80%)	0 (0%)
All cell types	238 (73.9%)	129 (54.2%)	4655 (93.5%)	171 (72%)	71 (55%)

*BF* Bonferroni, *FDR* false discovery rate

^a^The maximal sample size of any cell type is NS = 71 and SM = 64. DNA amount, or data quality eliminated some samples from analysis. Six subjects only have a WB sample, without any cell type

^b^Highly specific were > 10^4^ *p* value difference in one cell type relative to all other cell types

^c^Replication comparison from the following references [13, 33–39]

**Fig. 1:**
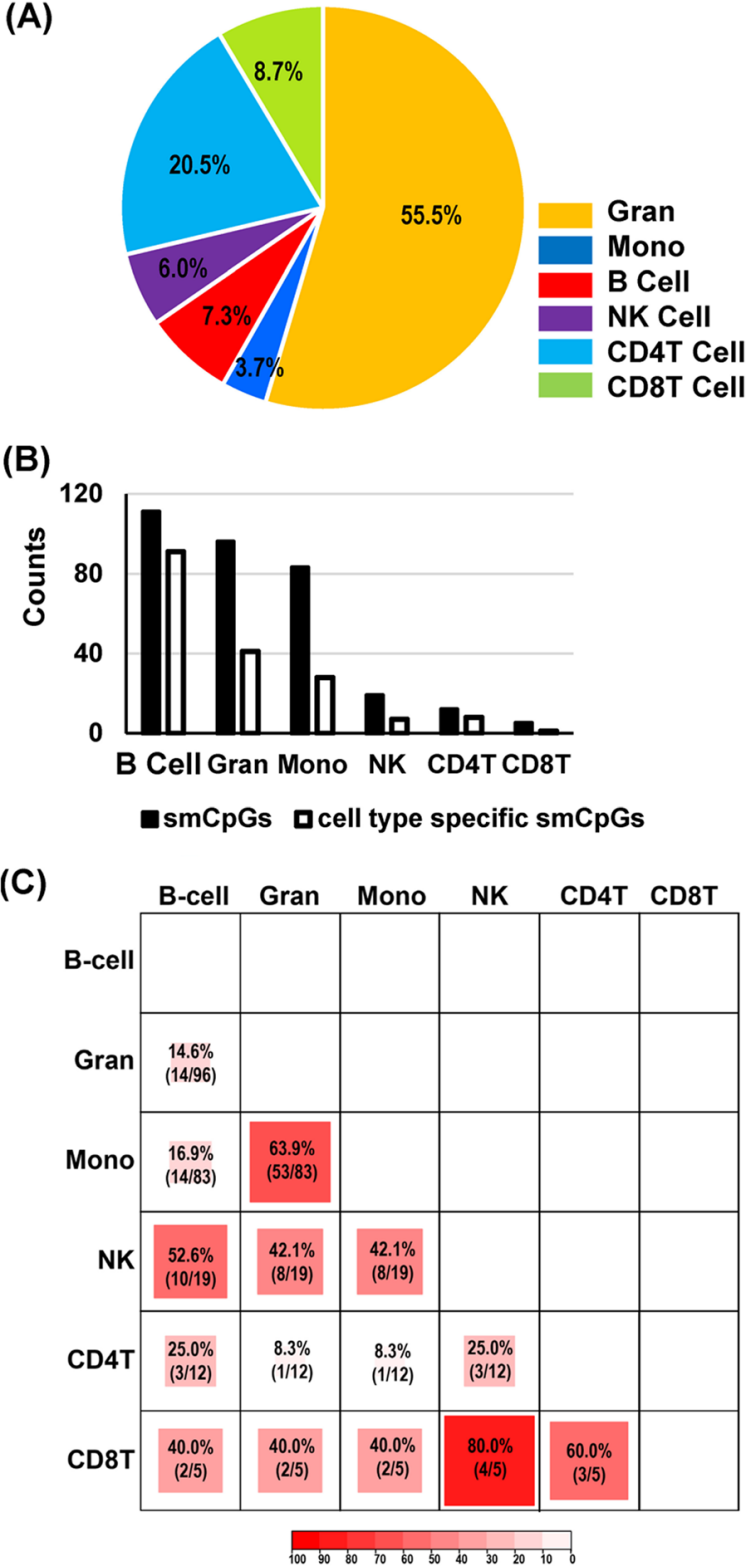
Blood cell types and detected smoking-associated CpGs (smCpGs). **A** The estimated average percentage of cell types in whole blood samples using Houseman model [18]. **B** Counts of smCpGs and cell-type-specific smCpGs observed at Bonferroni (BF) statistical significance among blood cell types. **C** Percent (counts) of overlap of BF significant smCpGs among blood cell types. Darker shading indicates greater overlap

At genome-wide (BF) significance, we identified a wide range in the number of smCpGs among cell types, from the least, 5 loci detected in CD8+ T cells (CD8T), to 111 loci in CD19+ B cells (B cells), although both CD8T and B cells are less than 10% of total leukocytes in normal blood samples (Table 1; Fig. 1A, B). In total, we identified 238 smCpGs from 6 cell types at BF cutoff (Additional file 7: Table S3), which were investigated thoroughly. When the significance threshold was changed from BF to FDR 5%, the number of smCpGs in B cells increased about 28-fold (3096 CpGs), while the increase in CD8T was only twofold to 10 CpGs (Table 1). Thus, smoking had a very different impact on DNA methylation across cell types, even among lymphocyte subtypes. Examining cell-type-specific CpGs, i.e., those that were only detected in a particular cell type at a given significance level, we found different degrees of specificity, ranging from 20% in CD8T to 82% in B cells at BF level (Table 1). At FDR 5%, the numbers of cell-type-specific smoking CpGs were increased for all cell types. Examining similarities between cell types, pairwise comparison of smCpGs detected at BF level (Fig. 1C) showed the myeloid cell types, granulocytes (Gran) and monocytes (Mono), shared 64% of smCpGs, whereas CD8T shared 80% (4/5 CpGs) with natural killer cells (NK cells). Only one CpG, cg05575921 in the *AHRR* gene, was common to all cell types at the BF level and this CpG is the most replicated smCpG in human blood DNA studies [32]. However, the effect size (methylation difference, nonsmoker–smoker, ΔMeth) and statistical significance levels of cg05575921 in each cell type were strikingly different, from the greatest effect (− 38%, *p* = 7.5 × 10^−38^) in Gran, to the least (− 4% *p* = 9.6 × 10^−8^) in CD8T cells. We examined the replication of the 238 cell-type smCpGs against a list of 8865 smCpGs identified previously at BF level in 10 published EWAS (study size range, *n* = 253 to 15,907) on smoking using whole blood samples [13, 33–40]. At genome-wide significance, 171 of 238 cell-type smCpGs (72%) were replicated in the EWAS literature, while the highly specific cell-type smCpGs (meaning at least 10^4^ *p* value difference in one cell type relative to all other cell types) showed a lower replication rate of 55% (Table 1).

**Fig. 2:**
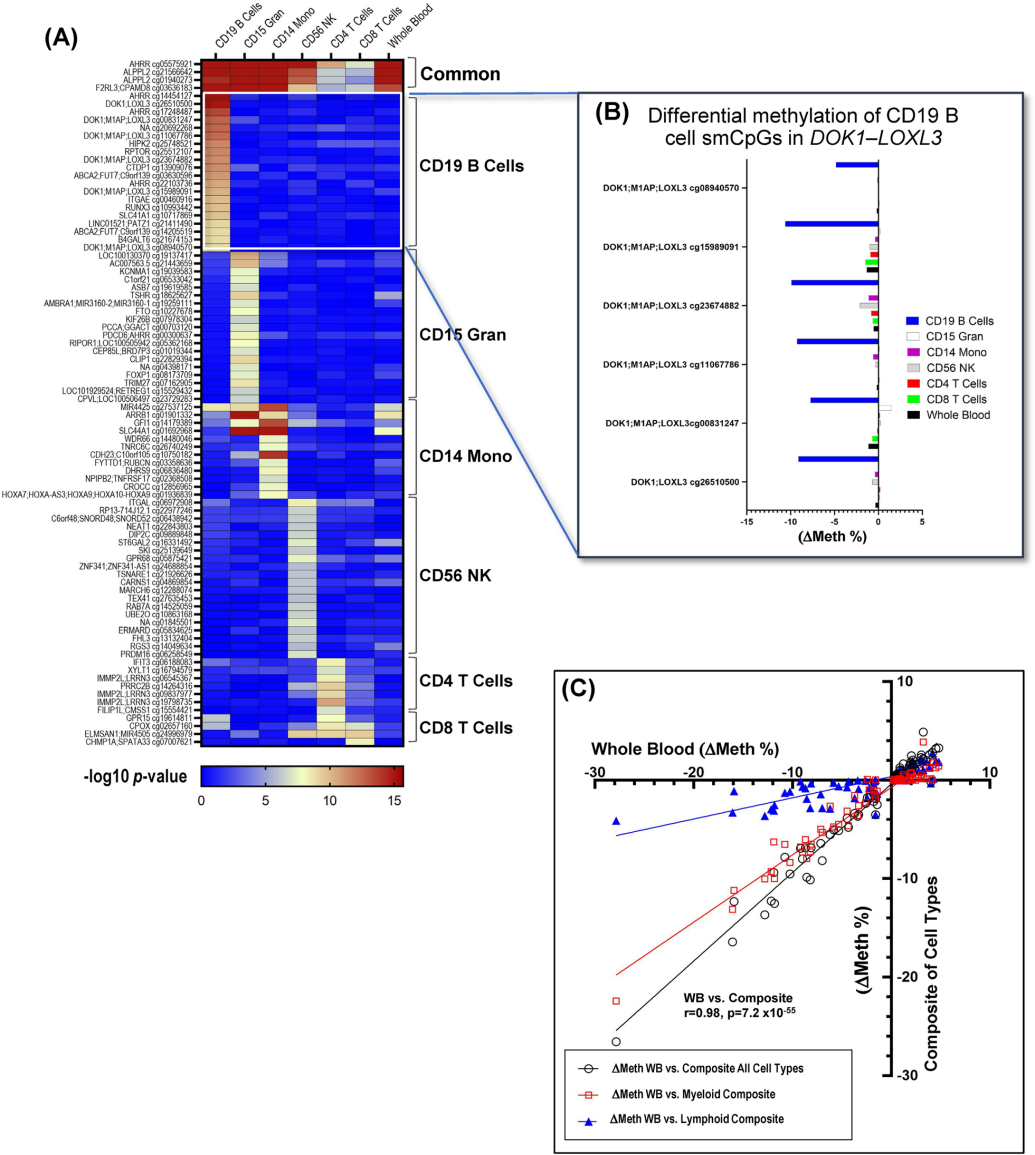
Blood cell types display unique smoking-associated methylation effects. **A** Heatmap displays significance level of highly cell-type-specific smCpGs across six cell types and whole blood. **B** Differential methylation level (ΔMethylation) across six cell types and whole blood shown for five B cell-specific smCpGs in the *DOK1-LOXL3* gene region. **C** Comparison of measured differential methylation of 74 whole blood smCpGs (BF significance) with estimated ΔMeth of composite whole blood, myeloid and lymphoid components

Comparing the directional change in methylation between smokers and nonsmokers in all cell types, 223 of 238 cell-type smCpGs (94%) had the same directional changes in 4 cell types or more; among these, 167 CpGs (75%) were hypomethylated and 56 (25%) hypermethylated in relation to smoking. In Fig. 2A, the top 4 rows are examples of smCpGs that were FDR significant in all cell types with the same directional change. However, as shown in Table 1, a large number of BF CpGs (54%) were highly specific and significant in only one cell type. A sample of the most significant of these highly specific smCpGs is displayed in the supervised heatmap (Fig. 2A). The heatmap also displays the significance level of these CpGs as assessed in a group of whole blood samples examined in this study; many showed only marginal (*p* < 0.05) significance in whole blood. In Fig. 2B, the effect size (ΔMeth) for a group of highly specific B cell smCpGs in the *DOK1-LOXL3* gene region is shown across all cell types. The large effect size in B cells contrasts with the very small ΔMeth for the other cell types and whole blood.

### Smoking-associated methylation changes in whole blood are the composite of methylation changes in cell types

We observed that smoking has different impacts on DNA methylation among isolated blood cell types. To confirm that smoking-associated effects in whole blood are a composite of individual cell-type effects measured separately, we selected 74 smCpGs that passed BF cutoff in whole blood to create composite ΔMeth values. To create the composite to compare to whole blood, for each cell type the average ΔMeth for each CpG was multiplied by the average cell-type proportion, then these six values were summed. That is for each CpG:

$$\begin{aligned} \triangle{\text{M}}\_{\text{composite}} & = \triangle{\text{M}}\_B\;{\text{cell}} \times 0.073 + \triangle{\text{M}}\_{\text{Gran}} \times 0.55 + \triangle{\text{M}}\_{\text{Mono}} \times 0.037 \\ & \quad + \triangle{\text{M}}\_{\text{NK}} \times 0.060 + \triangle{\text{M}}\_{\text{CD}}4{\text{T}} \times 0.20 + \triangle{\text{M}}\_{\text{CD}}8{\text{T}} \times 0.087 \\ \end{aligned}$$

In Fig. 2C, the ΔMeth_composite of all cell types is plotted against the measured ΔMeth for that CpG in the whole blood sample; the composites of myeloid and lymphoid cell types are also plotted against whole blood. Figure 2C shows the highly significant correlation (*r* = 0.98, *p* = 7.2 × 10^−55^) between the composite ΔMeth values and whole blood ΔMeth values at 74 smoking-associated CpGs. The slopes for myeloid and lymphoid composites provide a view of the relative contribution that these cell-type groups make toward smoking-induced methylation changes in whole blood.

### Subtype shift in isolated B cells associated with smoking

A recent method from Salas et al. [30] used reference DNA methylation profiles from 12 leukocytes subtypes (neutrophils, eosinophils, basophils, monocytes, naïve and memory B cells, naïve and memory CD4 and CD8 T cells, natural killer, and CD4 T regulatory cells) to deconvolute the relative abundance of these subtypes in whole blood. We used this model to detect smoking-associated subtype shifts (especially from naïve to memory) within each isolated cell type. Table 2 lists the cell-type frequency averages in smokers and nonsmokers and *p* values for differences after adjusting for age, sex, ancestry, and BMI. CD4T cells and CD8T cells isolated from smokers displayed a trend, shifting from naïve to memory, while the shift toward memory within the isolated B cells was significant (nonsmokers, naive B cells 60.0% of total CD19+ B cells; smokers, naïve B cells 52.8%; *p*adj = 0.033).

**Table 2:** Estimated proportion of leukocyte subtypes within isolated cell-type fractions from nonsmokers and smokers

Cell type	Subtype	Nonsmokers Percent within the isolated fraction	Smokers Percent within the isolated fraction	Difference	p*	*p*-adj**
		(Mean ± S.E.M.)	(Mean ± S.E.M.)	(Mean) (%)		
B Cell (CD19+)	Naïve B	60.0% ± 1.9%	52.8% ± 2.2%	− 7.20	0.014	0.033
	Memory B	31.3% ± 1.8%	36.0% ± 2.2%	4.70	0.096	0.12
Granulocyte (CD15+)	Neutrophils	95.0% ± 0.3%	93.6% ± 0.6%	− 1.40	0.028	0.054
	Basophils	0.1% ± 0.0%	0.2% ± 0.1%	0.10	0.134	0.348
	Eosinophils	0.0% ± 0.0%	0.0% ± 0.0%	0.00	0.334	0.26
Monocyte (CD14+)	Monocytes	93.8% ± 0.5%	93.1% ± 0.4%	− 0.60	0.332	0.684
NK Cell (CD56+)	Natural killer	49.4% ± 1.7%	46.5% ± 1.8%	− 2.90	0.244	0.231
CD4T Cell (CD4+)	Naïve CD4T	35.1% ± 2.0%	30.2% ± 2.0%	− 4.90	0.09	0.413
	Memory CD4T	49.2% ± 1.8%	52.1% ± 1.7%	2.90	0.253	0.739
	Regulatory T (Treg)	5.3% ± 0.4%	5.6% ± 0.5%	0.30	0.641	0.544
CD8T Cell (CD8+)	Naïve CD8T	37.6% ± 2.2%	31.3% ± 2.7%	− 6.40	0.07	0.319
	Memory CD8T	45.7% ± 2.0%	48.9% ± 2.4%	3.20	0.31	0.648

Method of Salas et al. [30] used to estimate subtypes within isolated cell types

*The *p* values are from linear regression of subtype proportion on smoking status only

**The *p*-adj values are linear regression *p* values after adjusting age, ancestry, sex and body mass index

To further assess if this subtype shift in B cells associated with smoking was a general feature in whole blood, we re-analyzed our previously published smoking EWAS dataset (GSE85210) [40] to estimate B cell subtypes in whole blood from 253 individuals (172 smokers and 81 nonsmokers). We confirmed that smoking was associated with a significant shift from naïve B cells to memory B cells (nonsmokers, naïve B cells were 79.2% of total B cells and decreased to 66.9% of total B cells in smokers; a loss of -12.3%, *p*.adj = 9.3 × 10^−7^; Additional file 7: Table S4) and these changes were significant after adjusting for age, sex and ancestry. Furthermore, using methylation levels of *AHRR* cg05575921 as a quantitative biomarker of smoking exposure we observed a significant dose–response relationship (*p*adj = 1.84 × 10^−6^) between smoking and naïve B cell subtype proportion (Additional file 2: Fig. S2). The original whole blood analysis of Su et al. [40] was adjusted for 6 cell types and identified 738 smCpGs at BF level. Following adjustment for 12 cell types, the number drops to 55, suggesting that the naïve-to-memory shifts in T and B cells may be important factors in other smoking studies.

### Functional annotation of smoking-associated CpGs

**Fig. 3:**
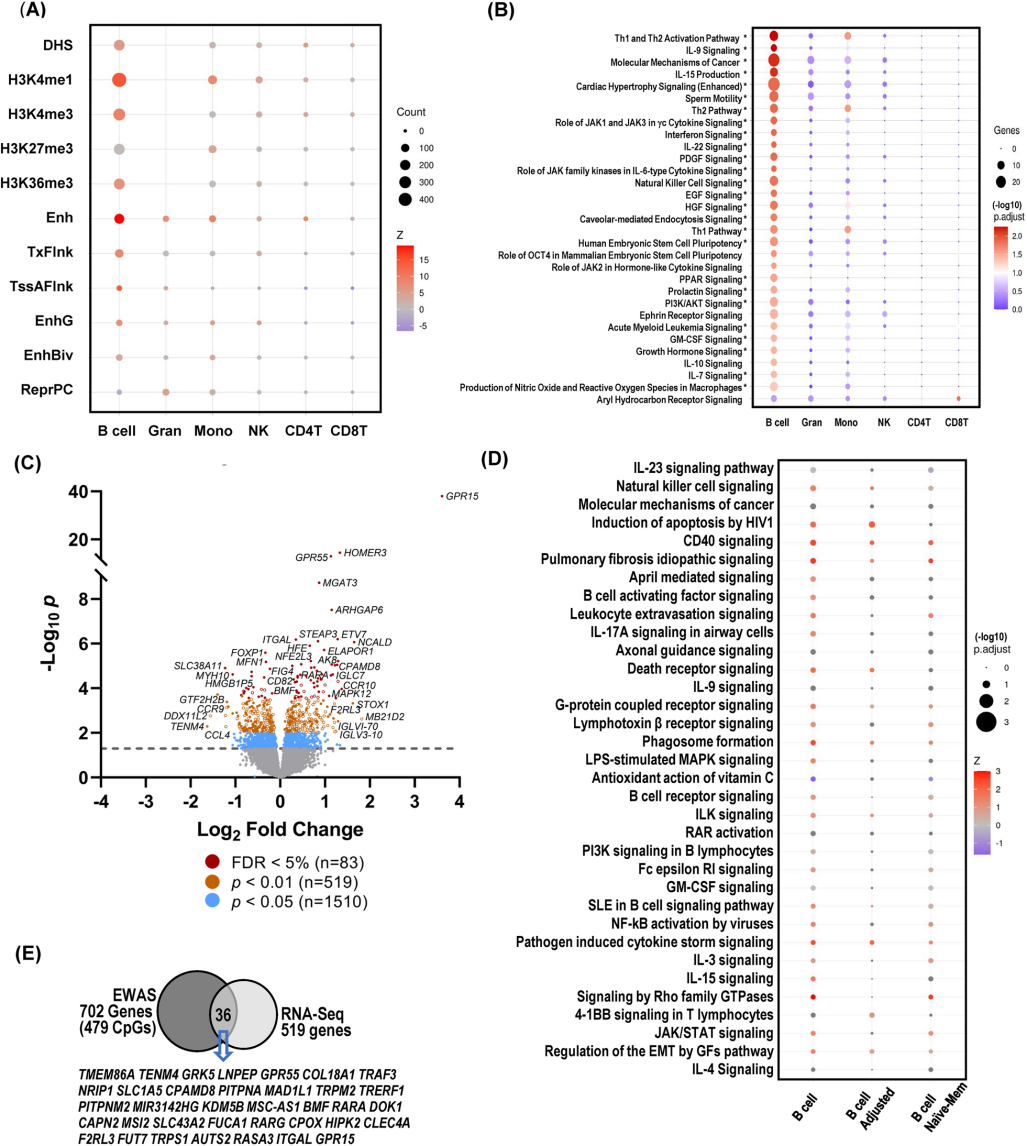
Enriched regulatory elements and pathways of smoking-associated CpGs (smCpGs). **A** cell-type-specific regulatory components. **B** canonical pathways of the genes annotated to smCpGs in each blood cell type. The color gradient indicts the significance (− Log_10_*p* value) and the size of dots indicts the number of genes involved. Detailed pathways and involved genes for B cell smCpGs are listed in Additional file 7: Table S6. *Indicates pathways of differentially methylated region-annotated genes involved in naïve-to-memory B cell differentiation. **C** Volcano plot of differentially expressed genes (DEGs) in B cells depicts negative Log_10_-transformed *p* values against Log_2_-transformed fold changes of DEGs in smoker B cells at different significance levels. Open circles indicate DEGs involved in naive B cell activation to be differentiated into memory cells. **D** Dot plot depicts the enriched pathways of blood B cell transcriptomics in smokers (*n* = 519 at *p* < 0.01), those remaining after adjustment (n = 419, *p* < 0.01) and those involved in naïve-to-memory B cell differentiation in smokers (*n* = 313, *p* < 0.01). Differentially expressed genes (DEGs) determined by RNA sequencing analysis (RNA-seq) with adjustment for nine covariates (age, sex, race, body mass index, CD4T %, CD8T %, NK %, Monocyte %, Granulocyte %) or ten (9 covariates + Naïve B cell %). Selected top-ranked canonical pathways are identified by IPA. Dot size indicts the –Log_10_ adjusted *p* values of the pathways. Dot color presents activation z-score trend. Detailed pathways and involved genes listed in Table S10. **E** Common 36 genes between RNA-seq (*p* < 0.01) and epigenome-wide association study (false discovery rate 1%) analyses of B cells in smokers

We next sought to investigate the biological relevance of the identified smCpGs by performing functional annotation and ontology enrichment analysis on smCpGs observed at FDR 1% (9 genes to 702 genes per cell type). Using eForge2.0 [41], we found distinctive enrichment of cell-type-specific experimentally identified regulatory components (Fig. 3A) with transcriptional histone marks, H3K4me1 and H3K4me3, and enhancer chromatin states showing the greatest enrichment in B cells. Using Ingenuity Pathway Analysis (IPA), we observed strong cell-type-specific enrichment of canonical pathways with B cells showing the greatest enrichment (Fig. 3B; Additional file 7: Table S6). Enriched canonical pathways were related to immune response (Th1 and Th2 activation, signaling by interleukins IL-7, IL-9, IL-10, IL-15, IL-22, and interferon, and transcriptional regulation via transcription factors (AHR), peroxisome proliferator-activated receptor (PPAR) and octamer-binding transcription factor 4 (OCT-4), signaling by JAK tyrosine kinases (JAK1, 2, 3), signaling by growth factors including epidermal growth factor (EGF), platelet-derived growth factor (PDGF), and hepatocyte growth factor (HGF). B cell epigenome enrichment in the immune signaling pathway supported a shift of naïve B cells to memory B cells in smokers.

To further explore the shift from naïve-to-memory B cells and to identify smCpGs associated with the shift, we carried out the B cell smoking EWAS both with and without adjustment for the estimated proportions of naïve and memory B cells based on the Salas et al. model [30]. This produced a dramatic change in the numbers of smCpGs and genes (from 606 smCpGs in 702 genes without adjustment to 163 CpGs in 173 genes with naïve and memory B cells adjustment) at FDR 1% (Additional file 7: Table S5). Smoking-related genes (and smCpGs) that remained in the list following adjustment included many known smCpGs frequently identified in blood (e.g., CpGs in *AHRR, ALPPL2, GPR15, GFI1, F2RL3*) and most of the highly B cell-specific genes shown in Fig. 2A, B. Among the genes associated with naïve-to-memory shift (486 CpGs/585 genes) were many related to cytokine signaling pathways for B cell functions and activation, Th1/Th2 activation and hematopoietic cancers (Additional file 7: Table S7).

### Smoking-associated transcriptional effects in B cells, subtype shifts and functional annotation

To further explore transcriptional changes associated with smoking and the shift from naïve B cell to memory B cells we carried out bulk RNA-seq on RNA from isolated peripheral blood B cells, a mixture of naïve and memory B cells, in 17 nonsmokers and 18 smokers. We analyzed differential expression using DESeq2 implemented in Partek Flow (Chesterfield, MO) adjusting for 9 covariates (age, sex, race, BMI, CD4T%, CD8T%, NK%, Gran%, Mono%) in differentially expressed gene (DEG) Analysis 1 and for 10 covariates in DEG Analysis 2 adding adjustment for naïve B cell proportions.

DEG Analysis 1 revealed 83 DEGs at FDR < 5% (Table 3) and 519 DEGs at *p* < 0.01 (Additional file 7: Table S8) between smokers and nonsmokers. The volcano plot (Fig. 3C) and supervised heatmap (Additional file 3: Fig. S3) display the significant DEGs between smokers and nonsmokers. DEG Analysis 2 adjusted for naïve B proportion and revealed genes strongly associated with smoking in B cells (Additional file 7: Table S9; 31 genes at FDR 5%; 419 genes at *p* < 0.01) independent of cell-type shifts. These 31 genes (FDR 5%) remaining at FDR significance are displayed as solid red dots in the volcano plot and include several known to be associated with smoking (e.g., *GPR15*; *GPR55, F2RL3*). We observed that a large number of genes were affected by the naïve adjustment (i.e., they lost significance, open circles in Fig. 3C) and this suggests they may have a role in naïve-to-memory B cell differentiation in smokers (313 out of 519 DEGs at *p* < 0.01, Additional file 7: Table S8). Importantly, many of the pathways of these 313 DEGs altered by naïve adjustment are related to B cell functions involved in differentiation to memory subtype (Additional file 7: Table S10; “Naïve-Mem” column of Fig. 3D). More specifically, pathways included B cell homing (e.g., *GPR15*, *ICAM1*), activation and class switching recombination (e.g., *CCL4*, *AICDA*, *RARA*, *PARP3*, *PIK3R6*, *VAV3*), memory cell gene expression (e.g., *TRERF1*, *MIR181A1HG*, *MSC-AS1*), and antibody-mediated humoral immunity (e.g., *IGHE*, *CCL4*, *ICAM1*, *CD226*).

**Table 3:** Representative peripheral blood B cell genes differentially expressed in smokers

Gene	Description	FDR	FC	Functions and ontologies
*GPR15*	G protein-coupled receptor 15	1.35E−34	12.20	Anti-inflammatory, chronic smoking biomarker
*HOMER3*	Homer scaffold protein 3	2.69 E−11	2.51	Negative regulation of T-cell activation, NFAT inhibition
*GPR55*	G protein-coupled receptor 55	5.52 E−10	2.19	Cannabinoid receptor, nicotine use disorder
*MGAT3*	Beta-1,4-mannosyl-glycoprotein 4-beta-N-acetylglucosaminyltransferase	6.90 E−06	1.83	Glycoprotein oligosaccharide biosynthesis, N-glycosylation
*ARHGAP6*	Rho GTPase activating protein 6	9.08 E−05	2.22	Actin cytoskeleton, cell morphology
*ETV7*	ETS variant transcription factor 7	1.37 E−03	2.44	Transcriptional repressor
*ITGAL*	Integrin subunit alpha L	1.37 E−03	1.28	ICAM receptor, leukocyte adhesion and transmigration, lymphopoiesis, lymphocyte-mediated cytotoxicity
*NCALD*	Neurocalcin delta	1.39 E−03	3.13	Calcium ion binding, cytosolic neuronal calcium sensor
*STEAP3*	STEAP3 metalloreductase	1.39 E−03	1.79	Erythrocyte iron homeostasis
*HFE*	Homeostatic iron regulator	1.80 E−03	1.58	Iron homeostasis, homology with MHC class I
*FOXP1*	Forkhead box P1	3.11 E−03	− 1.26	Transcriptional regulator, B cell differentiation
*AK8*	Adenylate kinase 8	6.26 E−03	2.44	Transfer terminal phosphate group to nucleoside
*NFE2L3*	NFE2 like bZIP transcription factor 3	6.26 E−03	1.60	Erythrocyte-specific globin gene expression, lymphoma
*MFN1*	mitofusin 1	6.39 E−03	− 1.25	Mitochondrial clustering and fusion
*AICDA*	Activation induced cytidine deaminase	7.10 E−03	2.22	B cell somatic hypermutation, gene conversion, and class-switch recombination
*CPAMD8*	C3 and PZP like alpha-2-macroglobulin domain containing 8	7.10 E−03	2.33	Complement, innate immunity
*SLC46A1*	Solute carrier family 46 member 1	7.10 E−03	1.38	Proton-coupled folate transporter
*TRERF1*	Transcriptional regulating factor 1	7.87 E−03	1.69	Transcriptional activator of CYP11A1
*MYH10*	Myosin heavy chain 10	1.22 E−02	− 2.09	B cell development, proliferation, and antibody response
*IGLC7*	Immunoglobulin lambda constant 7	1.23 E−02	2.18	Immunoglobulin light chain production
*L1CAM*	L1 cell adhesion molecule	1.23 E−02	1.50	Neuronal migration and axonal growth, brain development
*RARA*	Retinoic acid receptor alpha	1.32 E−02	1.31	B cell growth and class-switch, T cell immunity
*TRPS1*	Transcriptional repressor GATA binding 1	1.32 E−02	− 1.28	Cell cycle, chondrocyte proliferation, breast cancer marker
*BBC3*	BCL2 binding component 3	1.57 E−02	1.61	Apoptosis, regulation of memory B cell survival
*MAPK11*	Mitogen-activated protein kinase 11	1.66 E−−02	1.78	MAPK signal transducer, inflammation, T cell immunity
*CD82*	CD82 molecule	1.75 E−02	1.25	CD4/CD8 associated, TCR/CD3 pathway costimulation
*MTARC2*	Mitochondrial amidoxime reducing component 2	1.83 E−02	1.68	N-oxygenated molecule reduction, drug metabolism
BMF*	Bcl2 modifying factor	2.20 E−02	1.38	Apoptosis, prevent autoimmunity, B cell homeostasis
*CCR10*	C–C motif chemokine receptor 9	2.92 E−02	2.59	Homing and migration of B cells, CCL27 receptor
ABCB4*	ATP binding cassette subfamily B member 4	3.00 E−02	− 1.33	Biliary lipid secretion, B cell adaptive immune response
MYL9*	Myosin light chain 9	3.40 E−02	1.95	T cell CD69 ligand, lung inflammatory immune response
*TICAM1*	Toll like receptor adaptor molecule 1	3.41 E−02	1.29	TLR adaptor, innate immunity against pathogens, anti-viral
ARHGAP32*	Rho GTPase activating protein 24	3.65 E−02	− 1.54	Neuronal cell differentiation
*RARG*	Retinoic acid receptor gamma	3.65 E−02	1.33	B cell growth and class-switch, T cell immunity
*MAPK12*	Mitogen-activated protein kinase 12	4.57 E−02	2.13	MAPK signal transducer, inflammation, muscle growth,
CD226*	CD226 molecule	4.62 E−02	1.69	Cytotoxicity, T cell proliferation, autoimmune diseases
PARP3*	Poly(ADP-ribose) polymerase family member 3	4.71 E−02	1.28	DNA damage repair, regulation of class switch recombination
*GPR132*	G protein-coupled receptor 15	4.86 E−02	1.26	Oxidized free fatty acid receptor, mitosis, hematopoiesis
ITGA3*	Integrin subunit alpha 3	5.61 E−02	1.55	Matrix degradation, endothelial cell migration
CCR9*	C–C motif chemokine receptor 10	8.03 E−02	− 2.29	T cell selection and migration, HIV-1 infection. CCL25 receptor
COL18A1*	Collagen type XVIII alpha 1 chain	1.38 E−01	1.86	Extracellular matrix, MAPK signaling, angiogenesis inhibition
IGHE*	Immunoglobulin heavy constant epsilon	1.66 E−01	2.32	IgE heavy chain constant region production
VAV3*	Vav guanine nucleotide exchange factor 3	1.68 E−01	− 1.22	Angiogenesis, B cell receptor activation and development
CCL4*	C–C motif chemokine ligand 4	2.15 E−01	− 2.31	B cell receptor pathway activation biomarker, CCR5 ligand

Differentially expressed genes (DEGs) determined by DESeq analysis between smokers (*n* = 18) and nonsmokers (*n* = 17) with adjustment for 9 covariates (age, sex, race, body mass index, CD4^+^T%, CD8^+^T%, natural killer cell%, monocyte%, granulocyte%)

Full DEG list (*n* = 519 at *p* < 0.01) including 83 genes at false discovery rate (FDR) < 5% in Additional file 7: Table S8

*FC* fold change in smokers compared to nonsmokers

*Genes involved in naïve B cell activation to be differentiated to memory cells (determined after adjustment with naïve B cell %)

### Comparison of smoking-associated B cell epigenome and transcriptome

We observed 36 genes in common between EWAS smCpG-annotated genes (FDR 1%) and smoking DEGs (*p* < 0.01) identified by RNA-seq (Fig. 3E; Additional file 7: Table S11). Most of these 36 genes show an inverse relationship between methylation and gene expression change suggesting epigenetic regulation of their gene expression. Among this group of genes, at least 15 (e.g., *CLEC4A*, *MSC-AS1*, *TRAF3*) are likely to be involved in naïve-to-memory differentiation (Additional file 4: Fig. S4). IPA indicated by this included early B cell development (e.g., IL-7 and BAFF signaling), regulatory and effector B cell functions (e.g., JAK/STAT, GM-CSF, IL-10, and IL-15 signaling), as well as migration to germinal centers (e.g., GPR and ILK signaling), activation and antigen presentation (e.g., PI3K and B cell receptor signaling). Furthermore, among this group were genes and pathways involved in B cell differentiation such as somatic hypermutation and class switching recombination (e.g., IL-9 and CD40 signaling), clonal expansion and fate decision (e.g., AhR and APRIL-mediated signaling), memory cell marker (CD27) signaling, immunoglobulin E allergic response (e.g., IL-9 signaling), and plasma cell functions and humoral immunity (e.g., RAR activation, IL-2 and NK cell signaling) (Additional file 4: Fig. S4). Overall, these epigenetic/transcriptomic changes support the observation of B cell subtype shift in smokers.

### SmCpGs are enriched among blood EWAS CpGs associated with human health

**Fig. 4:**
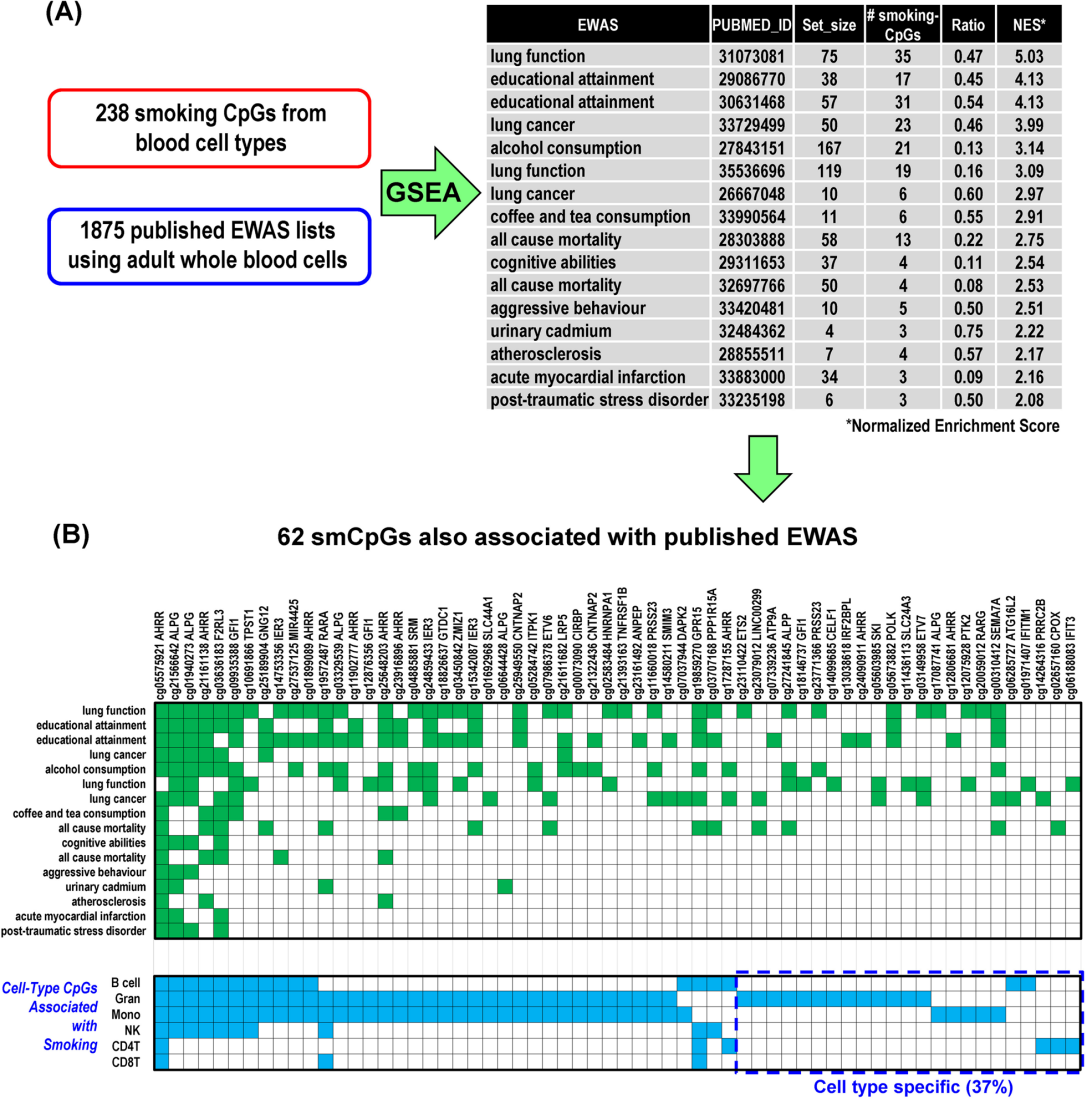
SmCpGs are enriched among blood epigenome-wide association study (EWAS) CpGs associated with human health. **A** Result of pre-ranked Gene Set Enrichment Analysis (GSEA) testing if smoking-CpGs were enriched among blood EWAS CpGs that associated with various health outcomes. All FDR *q* value < 0.003. **B** Distribution among cell types of smoking-CpGs associated with health outcomes

We performed Gene Set Enrichment Analysis (GSEA) [42] to test if cell-type smCpGs were overrepresented among published blood EWAS CpGs associated with various diseases and health-related phenotypes at the BF level. To this end, we retrieved CpG lists of blood EWAS from the EWAS catalog (1593 studies, [43]), the EWAS Atlas (202 studies, [44]), and our group curated 80 studies not in the catalog identified in the PUBMED database. Then, using the 238 cell-type smCpGs as the pre-ranked gene set and 1875 sets of CpGs curated from the published EWAS in human blood as the reference gene sets, we found smCpGs were overrepresented among 16 EWAS CpG sets associated with human health (Fig. 4A). It is noteworthy that all of these 16 EWAS adjusted for “smoking” as a covariate although there may be residual confounding. The most significant CpG set was from a “lung function” EWAS, which overlaps 35 cell-type smCpGs, with a normalized enrichment score (NES) 5.03 and a p-value (Fisher Exact) 8.8 × 10^−88^. In total, 62 cell-type smCpGs were also identified as EWAS CpGs related to human health. The upper panel of Fig. 4B displays the associated phenotypes (green blocks) and the lower panel provides the distribution of these smCpGs across cell types (blue blocks). However, we observed that the majority of the smCpGs (39 CpGs, 63%) identified in this enrichment were not cell-type-specific and most (54 CpGs, 87%) were significant in myeloid cells (monocytes and/or granulocytes).

### SmCpGs are linked to genetic variation associated with human diseases and health outcomes

Whether smoking-associated altered DNA methylation has any causal effect on human phenotypes has not been thoroughly evaluated. We performed an integrative analysis that uses data from multi-omics studies in large human populations to functionally link smCpGs to genetic variation associated with human phenotypes. The workflow is outlined in Additional file 5: Fig. S5. We first identified mQTLs that could serve as proxies for smCpG sites identified in this study. For this, we utilized single-nucleotide polymorphism (SNPs) associated with methylation of CpG sites in peripheral blood measured in four independent large studies on European ancestry participants: the Biobank-Based Integrative Omics Studies (BIOS, *n* = 3841) [45], the Framingham Heart Study (FHS, *n* = 4170) [46], a meta-analysis of the Lothian Birth Cohorts and the Brisbane Systems Genetics Study (LBC_BSGS, *n* = 1980) [47], and UK Household Longitudinal study (UKHLS, *n* = 1111) [48]. After filtering mQTLs (with *p* value < 2 × 10^−11^, distance of CpG to SNP < 10 kb, and replicated in two or more studies), we were able to proxy 137 smoking CpGs by using 2709 mQTL SNPs (Additional file 7: Table S12). To determine if these 2709 mQTL proxy SNPs were associated with GWAS phenotypes, we directly compared them with GWAS results and examined if they were in complete linkage disequilibrium (LD) with GWAS SNPs (~ 130,000 SNPs with *p* value < 5 × 10^−8^). We found 74 smCpGs linked with 579 mQTL SNPs that were either GWAS SNPs or in complete linkage disequilibrium (LD) with GWAS SNPs, which are associated with 4 categories of human phenotypes including lung function, disease risk, blood traits, and other traits (Additional file 7: Table S13).

**Fig. 5:**
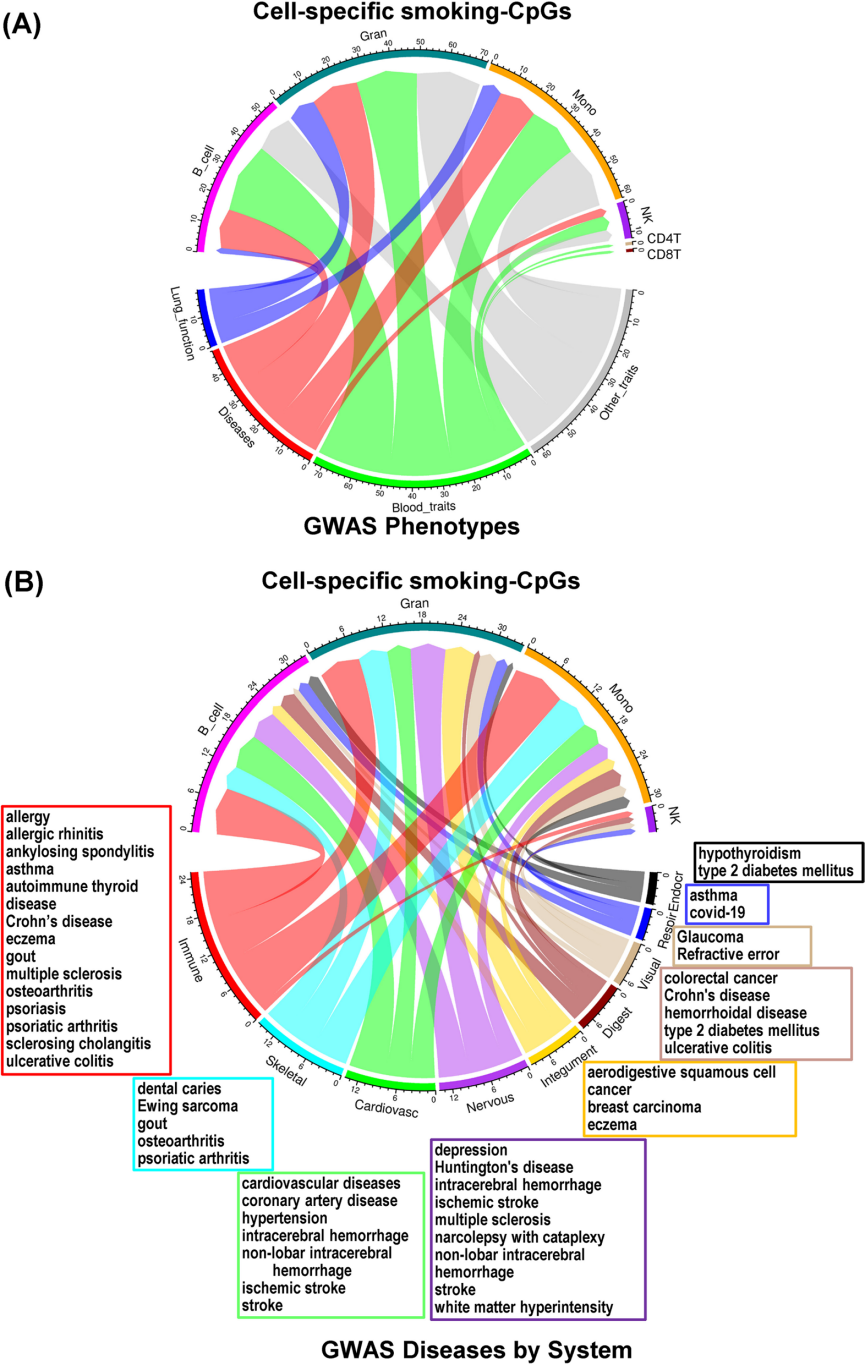
Comparison analyses of smoking CpGs with public data sets. **A** Cell-type-specific smoking CpGs were linked to genome-wide association study (GWAS) single nucleotide polymorphisms (SNPs) that associated with lung function, diseases, blood phenotypes and other traits. **B** Cell-type-specific smoking CpGs linked to disease categories grouped by anatomical system

Tracing the cell-type origin of these smCpGs (Fig. 5A), we found lung function traits (blue ribbon) mainly linked to myeloid cells (Gran and Mono); but disease risk (red ribbon), blood traits (green ribbon) and all other traits (gray ribbon) were linked to both B cells and myeloid cells (Fig. 5A; Additional file 7: Table S14). There were only very small number of smCpGs from NK or T cells linked to disease risk or phenotypes. We further visualized the connection of these smCpGs to GWAS disease categories grouped by the anatomical system (Fig. 5B; Additional file 7: Table S15). Among disease categories, “immune” had the most CpG-disease links (24 CpGs, red ribbon), which mainly originated from Gran, Mono and B cells, with a few from NK cells. Diseases in this category include allergy, asthma, ankylosing spondylitis, Crohn's disease, eczema, multiple sclerosis, osteoarthritis, psoriasis, psoriatic arthritis, sclerosing cholangitis, and ulcerative colitis. Similarly, Gran, Mono and B cells specific smCpGs were linked with cardiovascular diseases, including hypertension, intracerebral hemorrhage, non-lobar intracerebral hemorrhage, ischemic stroke and stroke.

### Integrating smCpGs with omics datasets

To further probe functional relationships, we utilized publicly available omics datasets generated from whole blood samples. We tested the association of smCpGs methylation levels with mRNA levels of nearby genes in *cis* (CpG position within a range from the transcription start site (TSS) − 10 kb to transcription ending site (TES) + 10 kb), termed as expression quantitative trait methylation (eQTM) analysis. After searching eQTM datasets generated from peripheral blood of BIOS samples (*n* = 3841) [45] and FHS samples (*n* = 4170) [49], we found 40 smCpGs associated in *cis* with mRNA levels at *p* < 1.0 × 10^−5^ (Additional file 7: Table S16, Column “eQTM”). Then we asked whether mQTL SNPs were eQTL SNPs that were associated in *cis* with mRNA levels (distance of CpG to SNP < 10 kb and CpG position within a range from TSS − 10 kb to TES + 10 kb). After searching 4 peripheral blood eQTL datasets (BIOS [45], FHS [46], CAGE [50], and eQTLGen [51]), we found 121 smCpGs (Additional file 7: Table S16, Column “eQTL”) that had 2,054 mQTL SNPs that were also *cis*-eQTL SNPs with *p* < 1 × 10^−5^.

We identified 36 smCpGs (Additional file 7: Table S16) which have significant signals in at least 4 omics datasets. Six smCpGs have significant signals across all the omics datasets (Additional file 6: Fig. S6), including cg03636183 (*F2RL3*), cg14753356 and cg15342087 (*IER3*), cg01971407 (*IFITM1*), cg03707168 (*PPP1R15A*), and cg00310412 (*SEMA7A*). The omics findings suggest a high priority for follow-up investigation of these smCpGs.

**Fig. 6:**
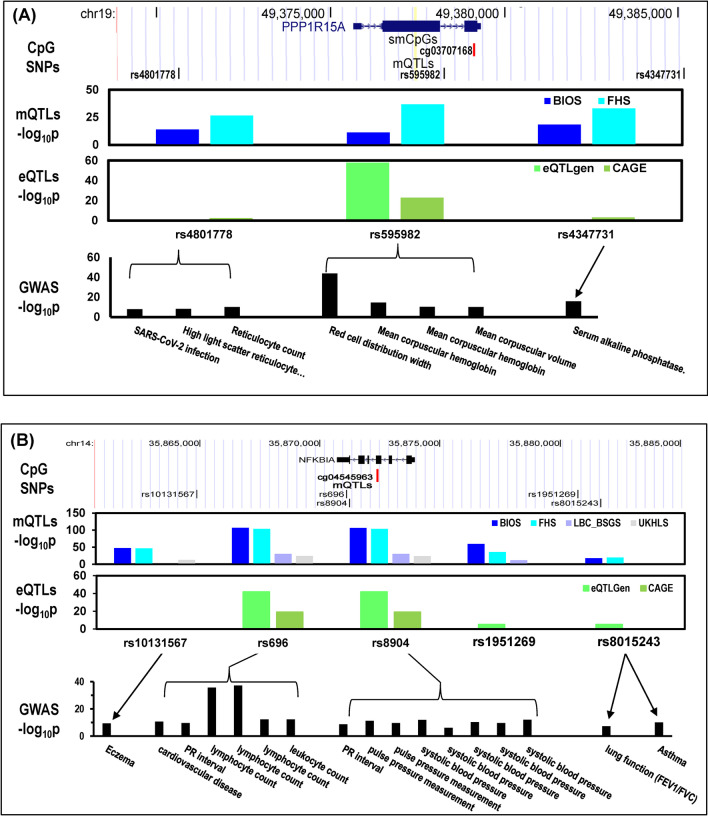
Examples of mechanistic linkage of epigenome-wide association study (EWAS) smCpG with disease risk. **A** SmCpG (cg03707168, red bar) in *PPP1R15A* exon 2 associated with COVID-19 infection and reticulocyte count via single nucleotide polymorphism (SNP) rs4801778, and other traits via rs595982 and rs4347731. **B** The B cell-specific smCpG in *NFKBIA* gene (cg04545963, red bar) linked via mQTL and eQTL SNPs (positions displayed by black bars) to GWAS of lung function, asthma, and several other disease risk and traits

An illustrative example of potential functional and mechanistic connections across these omics endpoints on the smCpG cg03707168 is shown in Fig. 6A. It maps in exon 2 of protein phosphatase 1 regulatory subunit 15A gene (*PPP1R15A,* also referred to as *GADD34*, Fig. 6A), which is an activating transcription factor 4 (ATF4)-target gene known to play a role in endoplasmic reticulum stress-induced cell death, and we previously reported it as a CD14+ monocyte-specific smCpG-annotated gene in atherosclerosis patients [28]. In omics datasets, its methylation level (mQTL) is associated with *PPP1R15A* mRNA level in BIOS study (i.e., a QTM with *p* = 1.4 × 10^−4^); it is also associated with SNPs (rs4801778, rs595982, rs4347731) which have been determined to be eQTL SNPs. Furthermore, this smCpG is also an EWAS CpG associated with lung function, all-cause mortality, and educational attainment (see Fig. 4B), all traits that are associated with smoking history. The relationship between methylation, gene expression and genotype provides a possible molecular mechanism linking this smCpG to smoking-related GWAS findings associating the gene to a variety of blood traits including mean corpuscular hemoglobin concentration, mean corpuscular volume, red blood cell distribution width, and reticulocyte count, and also susceptibility to COVID-19 infection.

Another important example includes a B cell-specific smCpG (cg04545963) in NF-κB inhibitor alpha gene (*NFKBIA*). cg04545963 has methylation levels associated with *NFKBIA* mRNA abundance (*p* = 5.7 × 10^−21^) in FHS samples (*n* = 4170) and genotypes of 4 nearby SNPs (Fig. 6B), which are also associated with gene expression levels (i.e., eQTLs). Intersecting these SNPs with GWAS studies links them to a large number of phenotypes and diseases including lung function (FEV1/FVC ratio), asthma, eczema, cardiovascular disease, systolic blood pressure, eosinophil count, and lymphocyte count (Fig. 6B).

## Discussion

Previously, as a pilot study, we identified distinct epigenetic effects of tobacco smoking among four leukocyte subtypes (CD2+ T cells, CD19+ B cells, CD14+ monocytes, and CD15+ granulocytes isolated with antibody-coated magnetic beads) in a small group of 10 smokers and 10 nonsmokers [40]. RRBS was also used to fine-map differential methylation in CD14+ monocytes [28]. The present study extends this work to additional cell types in a larger group of healthy adult smokers (*n* = 67) and nonsmokers (*n* = 74) to allow EWAS identification of cell-type-specific smoking effects.

We observed B lymphocytes display a large number of highly cell-type-specific alterations, suggesting possible differences in the mechanisms driving these events in lymphocyte lineages. In contrast, T cell subsets show very limited smoking-associated methylation effects. Both CD4+ and CD8+ T cells mature in the thymus and display a relatively long lifetime while remaining largely quiescent and proliferating only in response to antigenic stimulation. The small number of epigenetic alterations in T cells suggests that quiescent T cells may be protected from encountering the toxic agents in tobacco smoke. B cells follow a different trajectory, developing from common lymphoid progenitors in the bone marrow and maturing in lymph nodes and the spleen [52]. The large number of highly B cell-specific smCpGs suggests that smoking targets B cells after they have committed to the B cell lineage and reside in secondary lymphoid tissues or bone marrow.

As expected, a composite measure using methylation values from individual cell types (effect size by cell-type proportion) was highly correlated with measurements in whole blood (Fig. 2C). Myeloid lineage cell types (monocytes and granulocytes composing 40–70% of blood leukocytes) generally displayed the greatest magnitude and significance of smoking-associated methylation differences among blood cell types and that the response was similar to that detected in whole blood. The reason for the greater myeloid difference is not completely apparent. While some CpGs such as *AHRR* cg05575921 are strongly affected in all lineages, effects are much greater in myeloid lineages relative to lymphoid cell types. Reynolds et al. [27] showed that *AHRR* cg05575921 was highly significantly altered in the CD14+ monocytes of former smokers who quit > 20 years previously. These observations strongly suggest a common mechanism across cell lineages for some loci like *AHRR* that involves effects in hematopoietic stem and progenitor cells (HSPCs) in the bone marrow. The presence of *AHRR* demethylation in a population of bone marrow HSPCs would explain why these smoking-associated methylation effects persist for a very long time in former smokers and why they are observed in all lineages. It is possible that both location and proliferation are important in cell-type effects. Myeloid cells are constantly proliferating from progenitors in the bone marrow and have lifetimes measured in hours, the large proportion of myeloid cells affected may indicate that as myeloid progenitors differentiate, they may be exposed continuously to the agents in smoke that drive methylation alterations.

Most EWAS studies use the 6 cell-type Houseman model to adjust for potential cell-type shifts and we show this model is not sufficient to adjust for naïve-to-memory shifts. Using the 12 cell-type proportion estimator of Salas et al. [30] we observed that within the isolated B cell fraction (Table 2), smokers displayed a significant reduction in naïve B cells and an increase in memory B cells, and we confirm this in a larger whole blood smoking EWAS (Additional file 2: Fig. S2). Comparing regression analyses that were unadjusted or adjusted for naïve and memory proportions indicated that the majority of B cell smCpGs were in fact related to this shift. Interestingly most of the highly B cell-specific smCpGs (Fig. 2A, B) remained markedly significant even after adjustment for proportions of naïve and memory B cells, suggesting the highly specific smCpGs may be altered after B cell commitment but prior to B cell activation, somatic hypermutation, proliferation and memory B formation.

It is unclear if the mechanism driving the shift from naïve-to-memory B in smokers is directly due to tobacco smoke components or is an adaptive immune response resulting from the higher frequency of respiratory infections in smokers. These possibilities could be related. In tobacco smoke, the mixture of polycyclic aromatic hydrocarbons, reactive oxidants, particles, and other toxic components is known to be immunosuppressive, affecting immune system function. This impaired system may be more susceptible to respiratory infection and the adaptive response drives the naïve-to-memory shift. Pathway analysis supports this possible interaction, we see enrichment of type 1 interferons (e.g., IFN-α/β) signaling that might be associated with respiratory infections in smokers. IFN signaling enhances the antiviral function of adaptive immune cells through the promotion of antibody production by B cells and cytotoxic responses by T and NK cells [53].

B-cell-specific smCpGs in *NFKBIA,* which encodes the cytoplasmic inhibitor interrupting nuclear translocation of NF-κB (IκB), were linked to mQTLs with numerous GWAS traits (Fig. 6B) and also pathways such as peroxisome proliferator-activated receptor (PPAR), IL-10 signaling and reactive oxygen species production in macrophages. NF-κB regulates immune and inflammatory responses against diverse cellular stimuli such as stress, oxidants, and infection, and incorrect regulation of NF-κB signaling has been linked to cancer and various inflammatory and infectious disorders [54]. Defective IκB in mice led to improper B cell maturation, antibody production, and secondary lymphoid tissue development [55]. Pathway analyses of B cell smCpG-associated genes indicated that tobacco smoke may also influence cytokine signaling in B lymphocytes to skew or impair T cell-dependent immune responses as well as T cell-independent B cell functions (Additional file 4: Fig. S4). Among the affected cytokine signaling genes, IL-7 and IL-4 regulate early B cell lymphopoiesis and pro-B cell survival [56, 57]. Key attributes of increased memory cell subpopulation (i.e., naïve-to-memory cell differentiation) in smokers may enrich signaling through CD40, IL-9, and IL-4 for immunoglobulin (Ig) class switch recombination and Ig production in activated B cells and through RAR, IL-10, and IL-21 for clonal expansion and fate decision [58–61].

Smoking-altered transcriptomics further tracked with B cell subpopulation shift in the smokers. DEGs were highly enriched in pathways involved in B cell migration, activation, class-switching and Ig production (e.g., GPR, actin cytoskeleton, B cell receptor, PI3K, CD40, and IL-9 signaling), differentiation to memory cells and IgG production (e.g., APRIL, IL-10, RXR activation, and NK cell signaling). Several DEGs (e.g., *APBP2*, *COL4A4*, *COL18A1*, *HIPK2*, *GDPD5*, *RARA*, *TRERF1*) were reported to be expressed differentially in memory cells (class-switched CD27+ IgG+ or CD27+ IgA+) compared to naïve cells (CD27-IgD+) [62]. We revealed that in B cells *GPR15* was the most upregulated gene by smoking (12-fold upregulation, adjusted *p* = 1.35 × 10^−4^) and cg19859270 was also highly significantly demethylated (− 10% ΔM) by smoking. GPR15 mediates lymphocyte homing and migration [63] and has been a meaningful epigenetic biomarker in whole blood and in the T cells of smokers [64]. In addition, decreased methylation of *GPR15* (cg19859270) was a strong predictor of GPR15+ helper T cell increase in smokers who have *AHRR* (cg05575921) hypomethylation [65]. Enhanced *GPR15* expression in CD3+ CD4+ T cells was strongly associated with tobacco and cannabis smoking and smoking-induced inflammation [65]. When we compare epigenome and transcriptome of B cells, 36 DEGs were annotated to the significant smCpGs, including *GPR15*, *RORA*, transcriptional regulating factor 1 (*TRERF1*), TNF receptor-associated factor 3 (*TRAF3*), collagen type XVIII alpha 1 chain (*COL18A1*), and homeodomain interacting protein kinase 2 (*HIPK2*). The genes and pathways signatures in common between methylome and transcriptome (Fig. 3E; Additional file 7: Table S11) suggest smoking is altering epigenetic regulation of gene expression, affecting the B cell differentiation program.

We performed an integrative analysis that uses data from omics studies in large human populations to detect smCpGs that may mediate human phenotypes through genetic variation. As two illustrative examples, we identified that smCpG cg03707168 in *PPP1R15A* exon 2 was linked to COVID-19 infection and blood traits including mean corpuscular hemoglobin concentration, mean corpuscular volume, red blood cell distribution width, and reticulocyte count (Fig. 6A), and also that a B-cell-specific smCpG cg04545963 in *NFKBIA* gene was linked to lung function (FEV1/FVC ratio), asthma, eczema, cardiovascular diseases, systolic blood pressure, eosinophil count, and lymphocyte count (Fig. 6B). Our approach facilitates the interpretation of EWAS and GWAS loci in specific cellular contexts, suggesting mechanisms, and pointing to cell types for detection of phenotype-specific biomarkers.

## Limitations

While our study was able to reveal highly significant smoking-associated B-cell-specific effects, the current study lacked statistical power and resolution to identify effects in subsets of T cells. At this time, it was not possible to carry out additional RNA-seq transcriptomic studies, which could reveal interesting connections to smoking-associated phenotypes. We were able to associate about 30% of the smCpGs with genes containing GWAS SNPs; however, there were many smCpGs that could not be linked to human phenotypes, possibly due to stringent significance cutoffs, CpG to mQTL SNPs distances, and the limitations of public GWAS data.

## Conclusions

To our current knowledge, the study presented here is the most comprehensive dissection of smoking-associated DNA methylation changes in specific human blood cell types. Smoking impacts DNA methylation very differently among cell types, with many unique effects in B cells. We observe tobacco-smoke exposure was associated with shifting proportions of naïve and memory B cells, evidenced in both methylation and transcriptomics. Furthermore, using this dataset and publicly available epigenome and genome maps, we linked smCpGs with GWAS-identified risk loci. This integrative analysis provides a bridge to better understand smCpGs and GWAS results, enabling future studies to elucidate potential mechanisms in smoking-associated diseases.

## Methods

### Study populations

As part of the Epigenetic Biomarkers of Tobacco Smoke Exposure project, a group of healthy volunteers was recruited with written informed consent at the NIEHS Clinical Research Unit (protocol 10-E-0063) between March 2013 and January 2018 from Raleigh, Durham and Chapel Hill region of North Carolina using advertisements. Nonsmokers were defined by self-report as not having smoked > 100 cigarettes in their lifetime. Smokers reported their average daily cigarette consumption for the past 3 months. Serum nicotine/cotinine levels were measured by HPLC–MS (Quest, Inc.) as an indication of their smoking exposure status for all subjects. Demographic summary information of subjects is given in Additional file 7: Table S1. Six subjects only have a WB sample, without any cell type, the maximal sample size of any cell type is NS = 71 and SM = 64.

### Peripheral blood leukocyte subtype isolation

The workflow of the blood cell-type isolation is shown in Additional file 1: Fig. S1. Briefly, granulocytes were isolated directly from whole blood using CD15 Dynabeads (Invitrogen, Waltham, MA), magnetic beads covalently coupled with an anti-human CD15 antibody, according to the manufacturer’s protocol. Additionally, whole blood was layered on Histopaque-1077 Ficoll medium in Accuspin™ Tubes (Sigma-Aldrich, St. Louis, MO) and density gradient centrifugation was performed to isolate the mononuclear layer. Peripheral blood mononuclear cells (PBMCs) were then counted for viability and incubated with CD34 MicroBeads magnetic beads (Miltenyi Biotec, San Jose, CA). CD34 negative cells were collected in the flowthrough, recounted on a Cellometer Auto T4 Bright Field Cell Counter (Nexcelom, St. Lawrence, MA), and incubated with CD14 Dynabeads to isolate CD14+ monocytes according to the manufacturer’s protocol. Flowthrough (CD14-) cells were again counted and split by volume. One half of the cells were incubated with CD19 PanB Dynabeads for isolation of CD19+ B cells. The other flowthrough half was split in two, with half incubating with CD4 Dynabeads (for CD4+ T cells) and half incubating in CD8 Dynabeads (for CD8+ T cells). All flowthrough (CD19-, CD4-, or CD8- cells) was then combined and incubated with CD56 MicroBeads (Miltenyi Biotec) according to the manufacturer’s protocol to isolate CD56+ NK cells. RNA and DNA were isolated using the ALLPrep DNA/RNA/miRNA Universal Kit (Qiagen).

### Methylation analyses

Extracted DNA (100–500 ng) was bisulfite converted and applied to the Illumina Human Methylation 450k or EPIC BeadChip at the National Cancer Institute Center for Genomics Research to measure methylation at 450,000 or 850,000 CpG sites following the manufacturer’s instructions. The raw IDAT files of 450K and EPIC methylation arrays were read into R with the minfi package [66] separately; the combineArrays function in minfi was utilized to combine the two arrays’ data together based on their common (452,567) probes. Then, the data was preprocessed with background and dye bias correction using the preprocess Noob method [67]. The ChAMP package was used to do BMIQ normalization [68, 69]. The combat function in sva package [70] was used to do batch (“Sample_Plate”) correction on methylation array data. Prior to normalization, DNA methylation data were filtered based on these criteria: any samples having more than 5% probes that failed detection, all CpG probes on the *X* and *Y* chromosomes, probes containing SNPs with a minor allele frequency ≥ 1% (in EUR or AFR populations of the 1000 Genomes Project) within 5 nucleotides to the CpG site, and probes failing QC standards. We also removed 43,254 probes reported to hybridize to one or more non-target sites in the genome [71]. There were ~ 420,000 CpG probes remaining after exclusions. To investigate associations between smoking and DNA methylation, normalized and batch-corrected beta-values were transformed to log ratio, defined as log_2_[*β*/(1 − *β*)], and then fitted using robust linear regression [31] adjusted for potential confounders, including age, sex, race, BMI, and possible contaminant cell-type proportions, estimated using the method of Houseman et al. [18]. The Winsorize technique (https://www.rdocumentation.org/packages/DescTools/versions/0.99.44/topics/Winsorize) was used in an alternative EWAS analysis to test if outlier data points affected the resulting smCpGs. The method of Salas et al. [30] was used to detect shifts from naïve to memory among T and B cells. The raw IDAT files have been deposited in Gene Expression Omnibus (GEO) database under the accession number (GSE224807).

### Bulk RNA-seq

Aliquots of total RNA (250 ng) from peripheral blood B cells were used to generate poly-adenylated RNA libraries with TruSeq Stranded Total RNA Ribo-Zero Human Gold kit (Illumina, San Diego, CA). Samples were indexed with NEXTfle-96 RNA-seq Barcodes (Bioo-scientific, Austin, TX) and 75 bp paired-end sequencing was performed on NovaSeq 6000 platform using S1 flow cell (Illumina) in the NIEHS Epigenomics and DNA Sequencing Core Laboratory. FASTQ files containing 26–119 million raw sequencing reads were aligned to hg38 using STAR and gene counts were generated with featureCounts using the GENCODE version 39 annotation. Count matrix data were then imported to Partek Flow (Partek Inc., St. Louis, MO) and quantification of transcript expression and differential expression analyses were performed using DESeq adjusting for covariates as done in methylation (age, sex, race, BMI, 5 cell-type proportions, with or without naïve B cell proportions). DEGs were determined between smokers and nonsmokers with a cutoff for significance at *p* < 0.05 and/or FDR-adjusted *p* < 0.01. Controlled hierarchical cluster analysis by smoking status generated heatmaps showing a structure of DEG expression trends and partition of DEGs into different clusters using Partek Flow. RNA-seq raw data are deposited in GEO (accession number: GSE220113).

### EWAS datasets

Public available EWAS results were downloaded from the EWAS Catalog [43] (http://www.ewascatalog.org/, accessed June 1, 2022) and the EWAS Atlas [44] (https://ngdc.cncb.ac.cn/ewas/atlas, accessed June 1, 2022). We selected associations from EWAS studies using adult whole blood samples that had a *p* value < 0.05/450000 (450k array) or 0.05/850000 (EPIC array) and a population size > 100.

### GWAS datasets

GWAS datasets used were NHGRI-EBI GWAS Catalog [72] (accessed August 1, 2022), Lung Function GWAS (GCST007429, GCST007430, GCST007431, GCST007432) [73] (accessed August 10, 2020), and COVID19-hg GWAS meta-analyses Round 7 released April 8, 2022 (https://www.covid19hg.org/results/r7/). We selected associations which had a *p* value < 5.0E − 8 and a population size > 1000 in either discovery or replication phase.

### Datasets of mQTLs

Publicly available mQTLs results were downloaded from four independent large studies on European ancestry participants: BIOS (*n* = 3841) [45], FHS (*n* = 4170) [46], a meta-analysis of LBC_BSGS (*n* = 1980) [47], and UKHLS (*n* = 1111) [48]. We selected mQTLs which were in *cis* (distance of CpG to SNP < 10 kb), *p* value < 2.0 × 10^−11^, and significant in at least two datasets.

### Datasets of eQTMs

Publicly available eQTMs results were downloaded from two independent large studies on European ancestry participants: BIOS (*n* = 3841) [45], FHS (FHS, *n* = 4170) [49]. We identified eQTMs in *cis* (CpG position within a range from TSS − 10 kb to TES + 10 kb) at FDR < 0.05.

### Datasets of eQTLs

Publicly available eQTLs results were downloaded from four peripheral blood eQTL datasets: BIOS [45], FHS [46], CAGE [50], and eQTLGen [51]. We selected eQTLs in *cis* (distance of CpG to SNP < 10 kb and CpG position within a range from TSS − 10 kb to TES + 10 kb) at *p* value < 1.0 × 10^−5^.

### SNP genotyping and linkage disequilibrium data

The SNP genotyping data were downloaded from the 1000 Genomes Project’s Phase 3 v5b (http://ftp.1000genomes.ebi.ac.uk/vol1/ftp/release/20130502/). The LD between SNPs was calculated using the VCFtools (https://vcftools.github.io/index.html) and genotype data from European populations. Two SNPs are in complete LD if r2 > 0.95.

### Statistical methods

The open-source R program (https://www.r-project.org/) on the Linux platform was used for all statistical calculations.

## Supplementary Information

**Additional file 1: Figure S1**. The workflow of cell-type isolations.

**Additional file 2: Figure S2.** Association of cg05575921 methylation and naïve B cell proportion in smokers.

**Additional file 3: Figure S3**. Genome-wide gene expression analysis of blood B lymphocytes from smokersand nonsmokers.

**Additional file 4: Figure S4**. Epigenetic and transcriptomic events in smokers’ circulating B-cells associated with activation and differentiation into memory cells predicted by pathway analyses.

**Additional file 5: Figure S5**. Flowchart of the method for linking smCpGs to disease risk and other traits.

**Additional file 6: Figure S6**. Venn diagram of integration of omics results.

**Additional file 7:** Supplementary tables.

## Data Availability

The datasets generated and/or analyzed during the current study are available in GEO (https://www.ncbi.nlm.nih.gov/geo/query/acc.cgi?acc=GSE224807 DNA methylation, and https://www.ncbi.nlm.nih.gov/geo/query/acc.cgi?acc=GSE220113 RNA-seq). The R scripts are available from the corresponding author on request.

## Abbreviations

BF: Bonferroni
DEG: Differentially expressed gene
eQTL: Expression quantitative trait loci
eQTM: Expression quantitative trait methylation
EWAS: Epigenome-wide association study
FDR: False discovery rate
FEV1: Forced expiratory volume 1
FVC: Forced vital capacity
GEO: Gene expression omnibus
Gran: Granulocyte
GSEA: Gene set enrichment analysis
GWAS: Genome-wide association study
IPA: Ingenuity pathway analysis
LD: Linkage disequilibrium
Mono: Monocytes
mQTL: Methylation quantitative trait loci
NK: Natural killer
NIEHS: National Institute of Environmental Health Sciences
NIH: National Institutes of Health
smCpGs: Smoking-associated differentially methylated sites
SNP: Single nucleotide polymorphism
TES: Transcription end site
TSS: Transcription start site
WB: Whole blood
