# Supplementary figures and images for: Epigenomic profiling of isolated blood cell types reveals highly specific B cell smoking signatures and links to disease risk
Source: Clin Epigenetics. 2023 May 25;15:90. doi: 10.1186/s13148-023-01507-8 (PMC10211291; doi:10.1186/s13148-023-01507-8)

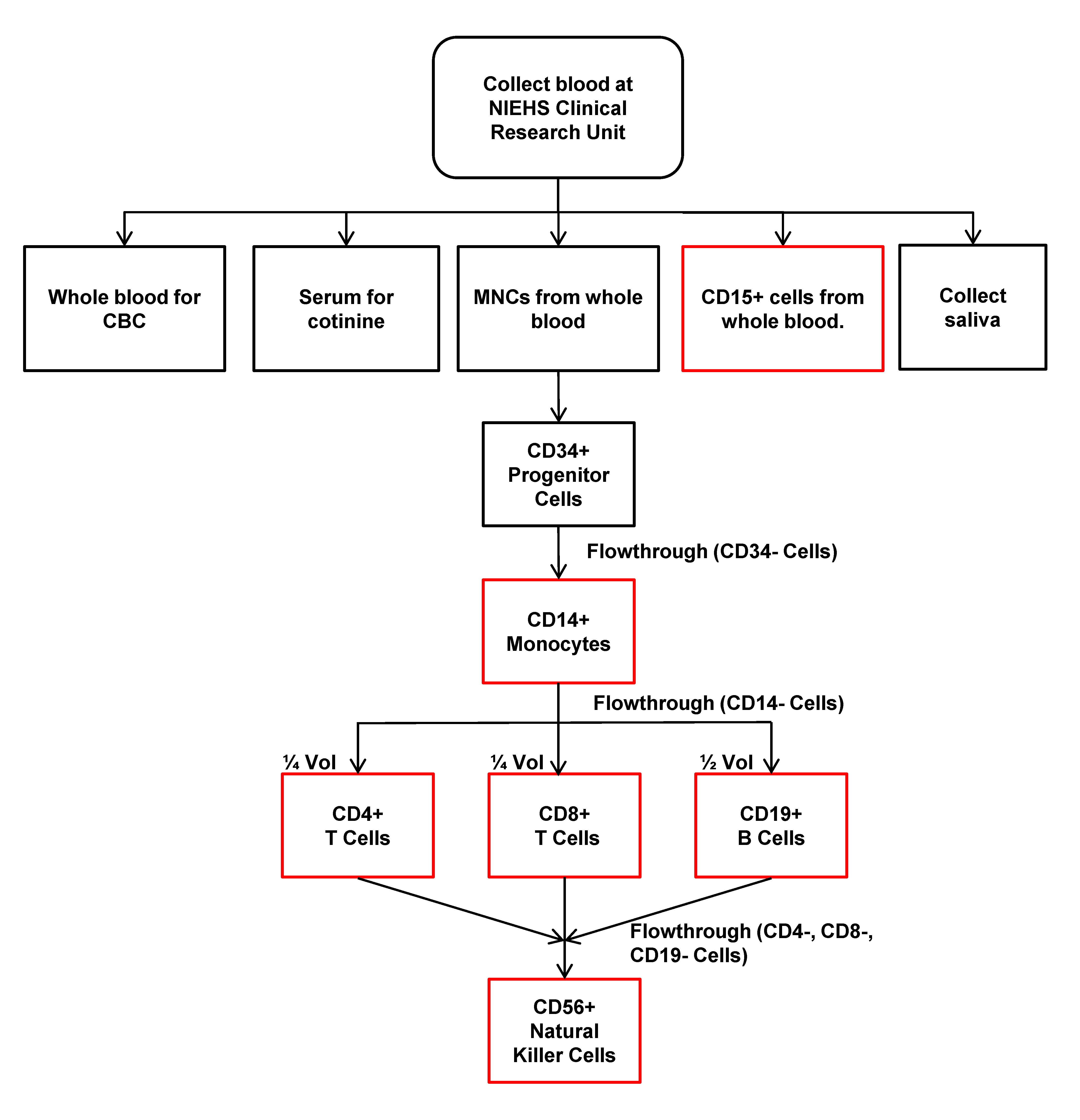

Supplement: Supplementary file 1 — Additional file 1: Figure S1. The workflow of cell-type isolations. [file 13148_2023_1507_MOESM1_ESM.jpg]

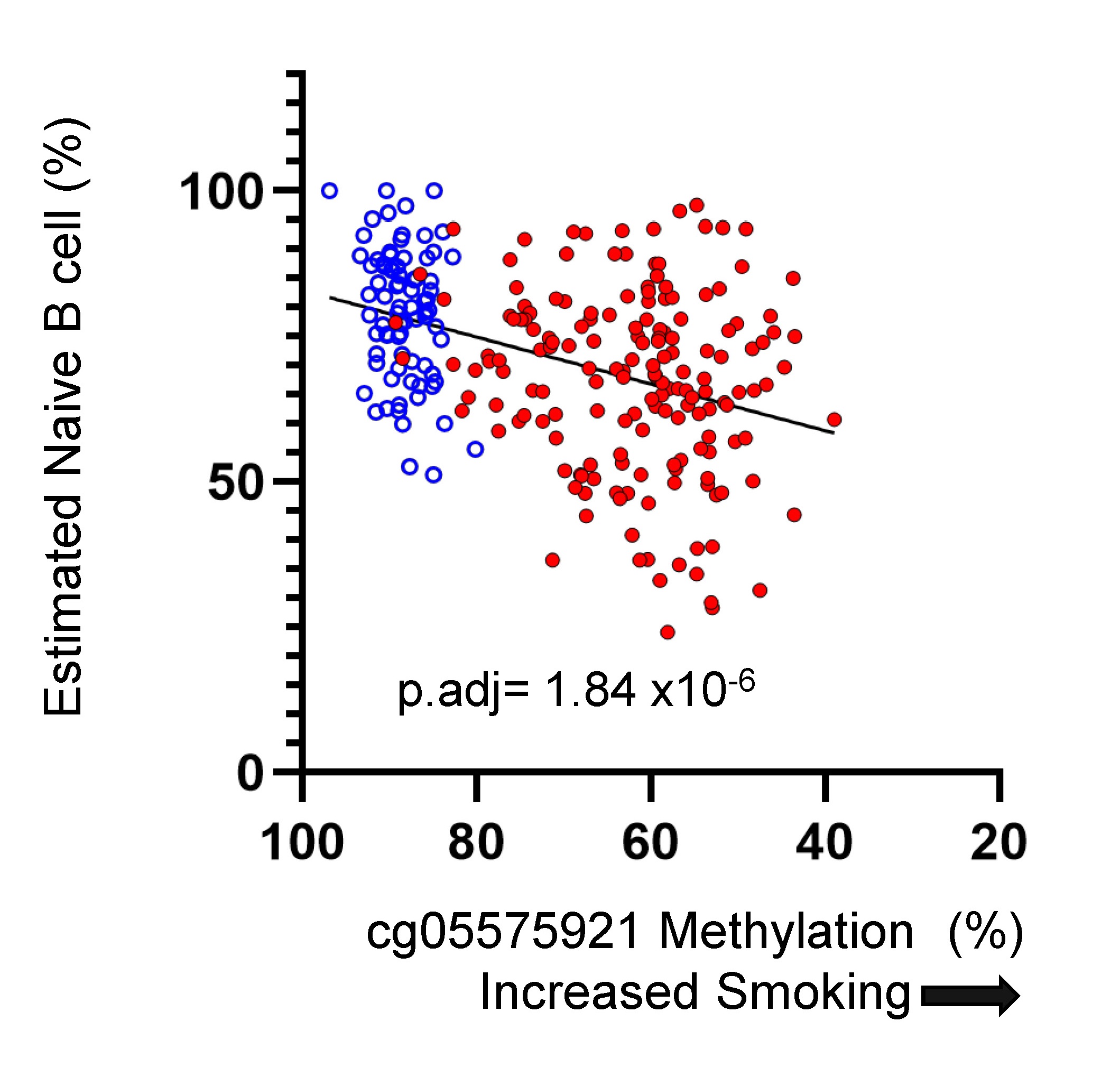

Supplement: Supplementary file 2 — Additional file 2: Figure S2. Association of cg05575921 methylation and naïve B cell proportion in smokers. [file 13148_2023_1507_MOESM2_ESM.jpg]

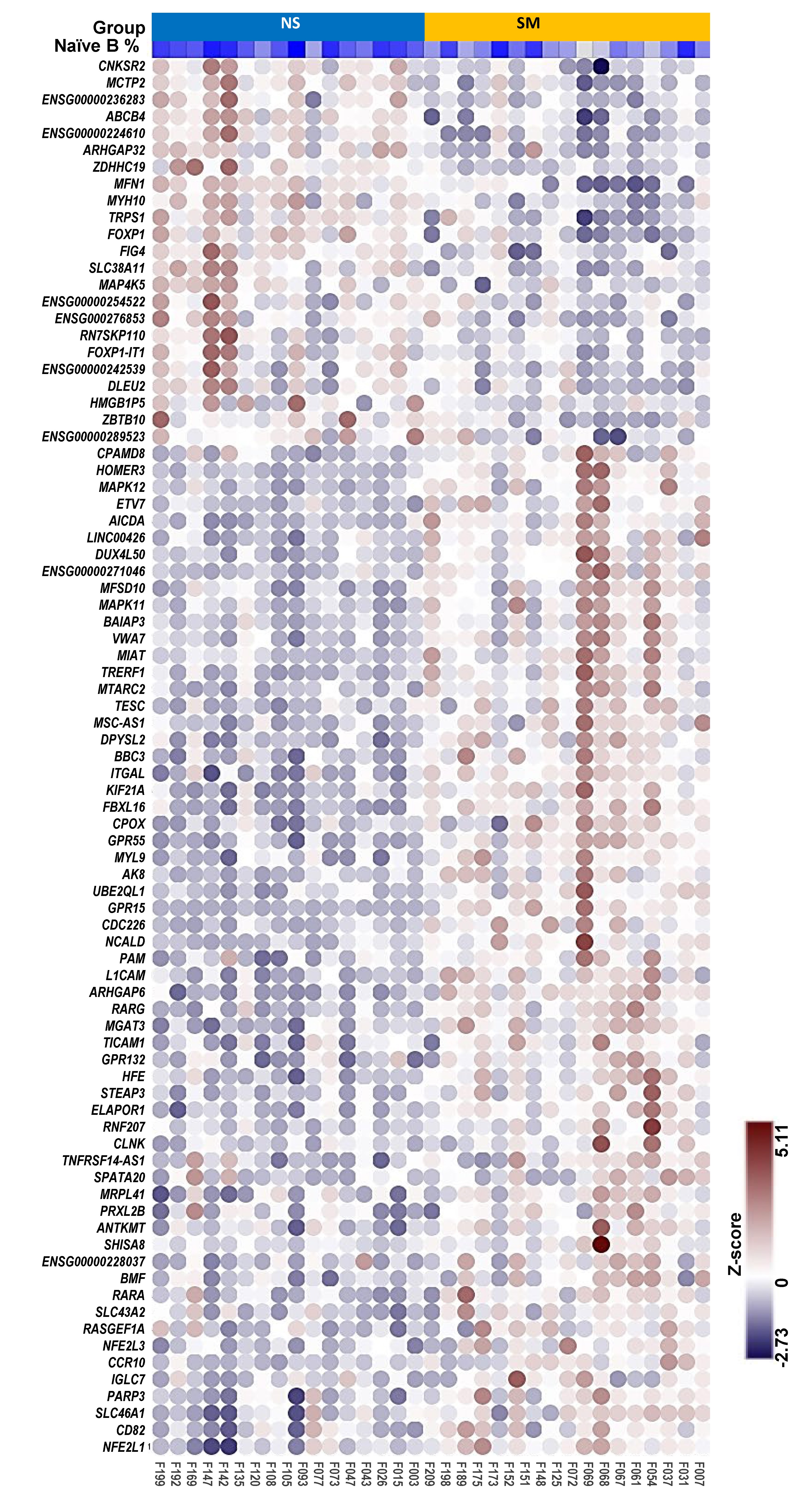

Supplement: Supplementary file 3 — Additional file 3: Figure S3. Genome-wide gene expression analysis of blood B lymphocytes from smokersand nonsmokers. [file 13148_2023_1507_MOESM3_ESM.jpg]

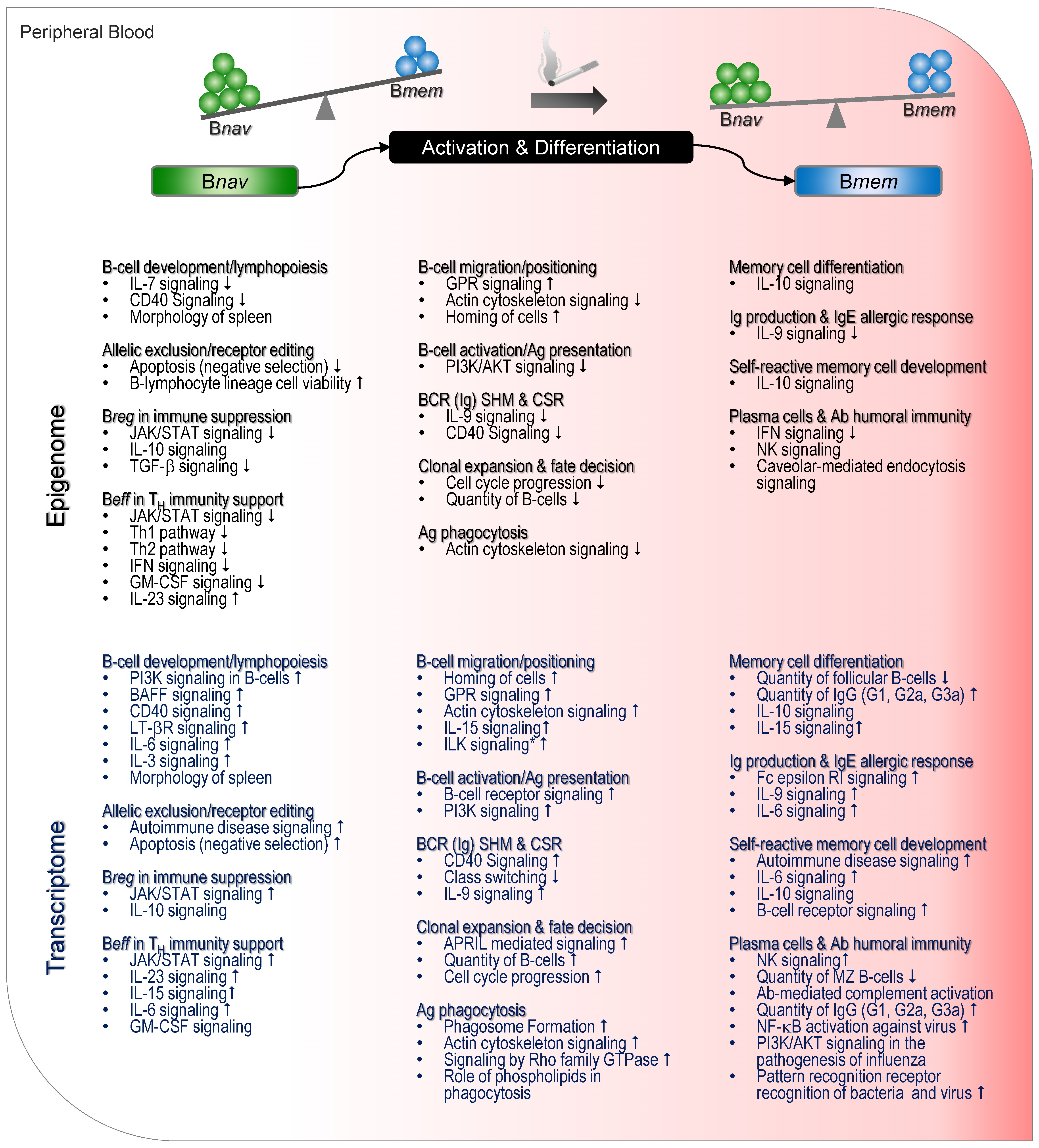

Supplement: Supplementary file 4 — Additional file 4: Figure S4. Epigenetic and transcriptomic events in smokers’ circulating B-cells associated with activation and differentiation into memory cells predicted by pathway analyses. [file 13148_2023_1507_MOESM4_ESM.jpg]

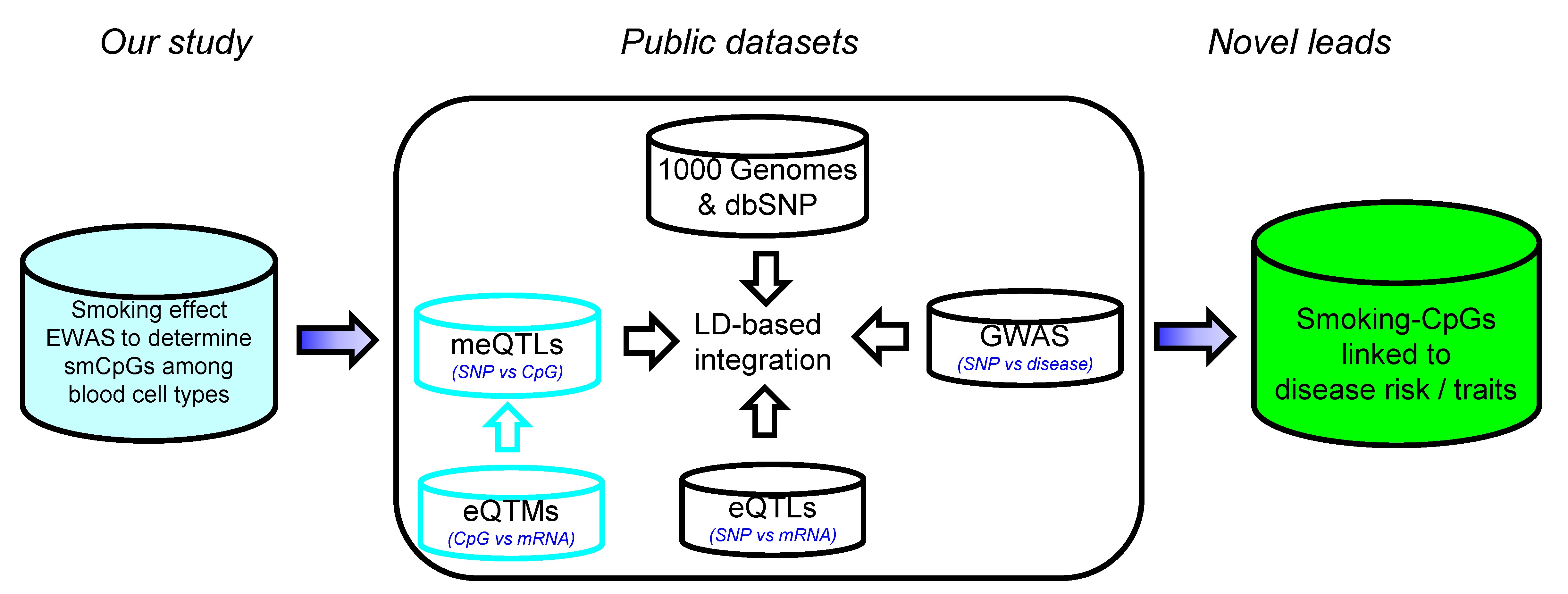

Supplement: Supplementary file 5 — Additional file 5: Figure S5. Flowchart of the method for linking smCpGs to disease risk and other traits. [file 13148_2023_1507_MOESM5_ESM.jpg]

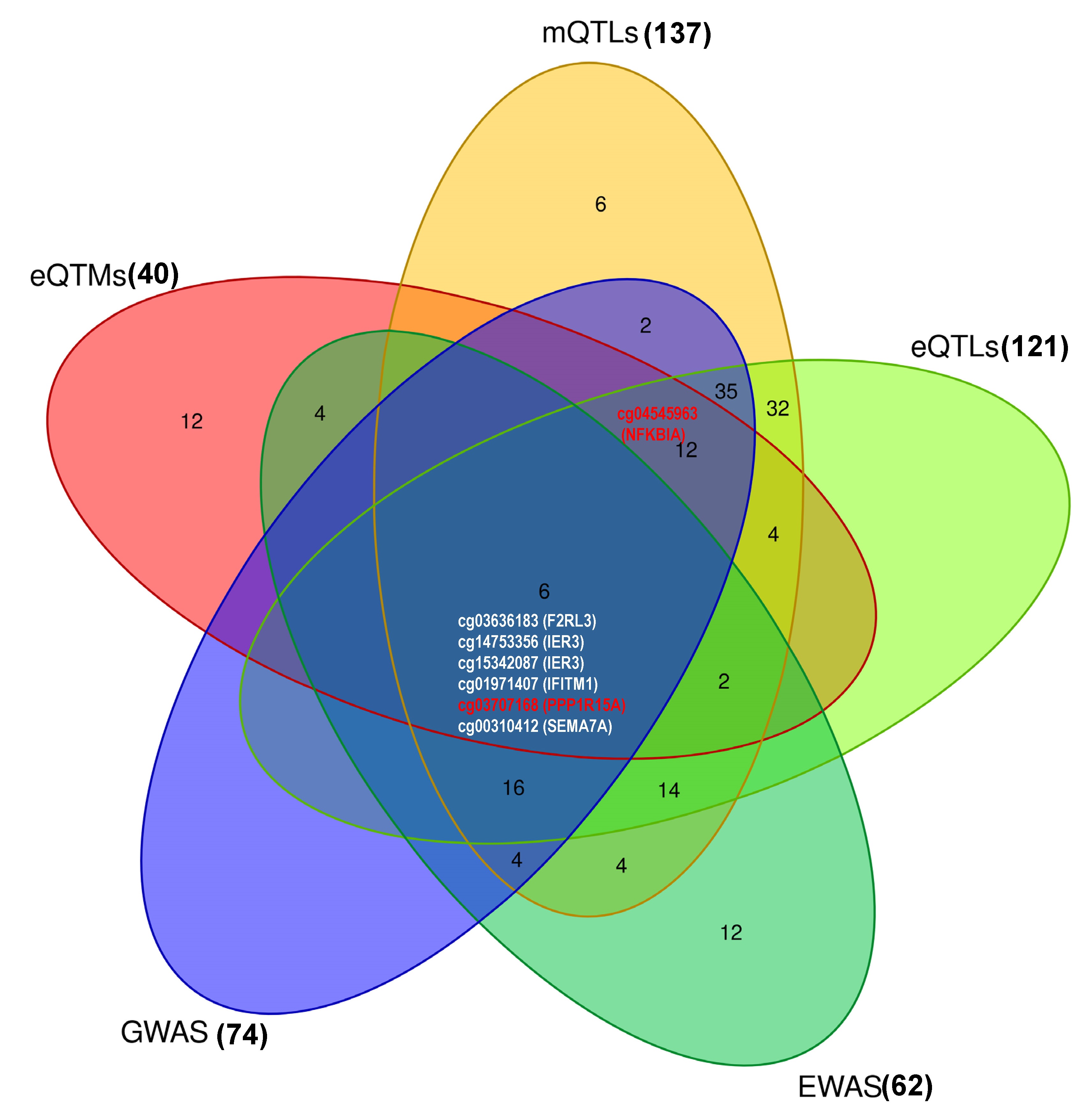

Supplement: Supplementary file 6 — Additional file 6: Figure S6. Venn diagram of integration of omics results. [file 13148_2023_1507_MOESM6_ESM.jpg]
